# How Do Collaborative Approaches Shape Power Dynamics in Therapeutic Relationships? Evidence From Recovery‐Oriented Mental Health Services

**DOI:** 10.1111/hex.70707

**Published:** 2026-06-02

**Authors:** Elena Faccio, Michele Rocelli, Lorenzo Binaggia, Elisa Giolli, Diego Romaioli, Giacomo Chiara, Claudio Agostini, Roberto Vitelli, Ludovica Aquili

**Affiliations:** ^1^ Department of Philosophy, Sociology, Education and Applied Psychology University of Padua Padua Italy; ^2^ University of Naples Federico II Napoli Italy; ^3^ Transmural Mental Health Department, Head of Psychiatry Unit – Northern District – Trento (Italy) Integrated University Healthcare of Trento Via Giovanni Battista Borsieri Trento Italy; ^4^ University of Naples Federico II. Department of Neurosciences and Reproductive and Odontostomatological Sciences University of Naples, Federico II Napoli Italy

**Keywords:** collaborative approach, dyadic interviews, mental health services, narrative analysis, power dynamics, recovery, therapeutic relationship

## Abstract

**Introduction:**

Recovery‐oriented mental health services emphasise collaboration, empowerment and service‐user involvement. However, less is known about how power is enacted within relationships described as collaborative. This study explores how power dynamics are interactionally produced, negotiated and made visible within recovery‐oriented therapeutic relationships.

**Methods:**

The study involved 15 service users from an Italian recovery‐oriented mental health service and 15 facilitators identified by them as significant to their recovery. Data comprised 30 individual interviews and 15 dyadic interviews. Dyadic interviews served both to gather participants’ accounts and to observe how the relationship was narratively enacted in interaction. Transcripts were analysed through a narrative‐interactional framework informed by Gubrium and Holstein's approach, with attention to narrative activation, composition, performance, collaboration and control. Coding focused on both what participants said about the relationship and how they co‐constructed, confirmed, redirected or constrained each other's narratives.

**Results:**

Four interpretive patterns were identified: parity‐oriented dynamics, declared parity with observed dominance, non‐categorizable dynamics, and acknowledged dominance. These patterns are better understood as heuristic configurations rather than definitive typologies. Parity‐oriented dyads were characterised by reciprocal narrative completion, mutual recognition and flexible turn‐taking. In other dyads, participants described the relationship as collaborative, while interaction showed more asymmetrical narrative control. One dyad displayed acknowledged directiveness, experienced by the service user as supportive rather than coercive. Across cases, professional role alone did not explain how power was enacted.

**Conclusion:**

Collaboration in recovery‐oriented relationships cannot be inferred from participants’ declarations or professional role categories alone. Rather, it needs to be examined through the situated interactional practices by which participants make space for, limit, complete or authorise one another's accounts. The study contributes a qualitative framework for analysing power as a relational and negotiated process within therapeutic relationships.

**Patient or Public Contribution:**

Individuals with lived experience were actively involved in interpreting the research findings through discussion and feedback sessions. A final dissemination meeting with participants enabled the validation of these interpretations and contributed to concluding reflections on the practical implications for mental health services.

## 1 Introduction

The World Health Organisation's Action Plan for Mental Health 2013‐2030 places significant emphasis on the active involvement of mental health service users, reflecting a paradigmatic shift toward participatory and person‐centred care. The WHO explicitly states: “People with mental health problems should be empowered and involved in mental health advocacy, policy, planning, legislation, service delivery, monitoring, research and evaluation” ([1], p.5). This involvement is not merely a procedural consideration but represents a fundamental reorientation of mental health services.

This paradigmatic shift raises fundamental questions about therapeutic relationships and how power is distributed within them. If service users are to be genuinely empowered and involved across mental health care, what happens to the traditional hierarchies that have long structured psychiatric and psychological services, and how can notions such as ‘collaboration’, ‘empowerment’ and ‘partnership’ be operationalised in relationships historically defined by significant power differentials? To contextualise today's emphasis on collaborative approaches, it is crucial to locate it within the broader history of the Recovery Movement – one of the most consequential transformations in mental health services over the past half‐century – challenging medical‐model assumptions about severe mental illness and the possibility of meaningful lives beyond diagnosis.

### 1.1 The Recovery Movement and Collaborative Approaches

Originating in the deinstitutionalisation efforts of the 1950s and 1960s, the recovery movement grew out of the civil rights movement, as people with mental distress sought to reclaim control over their lives, recover from the stigma of diagnosis, and resist mandatory treatment [2]. In subsequent decades, it gained momentum by contesting the dominant biomedical paradigm, diagnostic labels and linear treatment models [3]. These groups challenged the idea that professional expertise was the only legitimate knowledge in mental health, asserting lived experience as a valid form of expertise. A significant turning point occurred in the 1980s with the emergence of the contemporary concept of ‘recovery’: personal testimonies showed that meaningful recovery was possible even for people with diagnoses traditionally associated with poor prognoses [4]. This grassroots movement then gained institutional recognition, with the WHO incorporating recovery principles into its guidelines by 2000. Over time, recovery increasingly emphasised partnership: first by involving users in individual care decisions, and later, from the mid‐2000s onwards, through co‐production in service design and delivery. In this trajectory, peer support workers ‐ people with lived experience employed to support others ‐ have been pivotal in institutionalising experiential knowledge alongside professional training.

Contemporary recovery‐oriented practice aspires to broad forms of co‐construction, extending user involvement beyond clinical encounters to encompass planning, governance, research and evaluation. Yet empirical research has repeatedly documented tensions between recovery rhetoric and everyday practice: despite formal commitments to recovery principles, traditional power structures often remain substantially intact [5, 6]. This gap strengthens the case for investigating how power actually operates within relationships and organisations that claim to be recovery‐oriented. The question of power in therapeutic relationships has been approached from multiple theoretical perspectives, each offering distinct ways to describe its mechanisms and imagine how it might be transformed [7, 8, 9].

### 1.2 Theoretical Perspectives on Power

Traditional medical‐model approaches to mental health care have long been marked by pronounced power asymmetries: the professional is positioned as the expert with specialised knowledge of mental illness and treatment, while the patient/service user is positioned primarily as a recipient expected to comply with recommendations. Research suggests that these asymmetries persist even in contemporary services. Pilnick and Dingwall's [10] critical review, ‘On the remarkable persistence of asymmetry in doctor/patient interaction’, shows how power imbalances continue to shape consultations despite decades of patient‐centred rhetoric. Likewise [11], study, ‘Paternalism, autonomy and reciprocity: ethical perspectives in encounters with patients in psychiatric in‐patient care’, found that paternalism remained the dominant professional stance despite normative trends toward reciprocity and autonomy. Taken together, these findings imply that shifting power in therapeutic relationships requires more than endorsing collaboration: it calls for deeper changes in how professional roles are conceptualised and enacted, and in the organisational cultures that sustain them. These findings also reveal the epistemological challenge of studying power itself. Power in therapeutic encounters is difficult to examine precisely because it operates less through explicit statements than through the manner of communication: discursive strategies, conversational sequences and interactional positioning that shape what becomes sayable, hearable and actionable within clinical dialogue.

The construct of ‘power’ is therefore irreducibly complex and situational, bound up with tacit rules that permeate everyday contexts and requiring investigative approaches flexible and nuanced enough to grasp the reality‐constructing work accomplished through discourse. Social constructionist approaches [12, 13, 14] offer a suitable framework for analysing these dynamics: power is not a fixed attribute held by individuals or roles, but a dynamic process continuously negotiated and constructed in interaction. In line with this perspective, and drawing on Plummer [15, 16], power within social interaction is not conceived as a static attribute or as a resource possessed by particular individuals or institutions. Rather, it is understood as a relational and ongoing process that circulates through interaction, simultaneously constraining and enabling, limiting and generative. From this perspective, the relationship is not merely the site in which power is exercised, but the arena through which it is negotiated, reconfigured and potentially redistributed, thereby constituting an indispensable analytical and practical point of departure.

Previous studies have examined this complexity from different perspectives. For example, Ribeiro et al. [17] and Ryttinger et al. [18] used the Therapeutic Collaboration Coding System to explore moments of change and their relation to collaborative processes, while Martinez et al. [19] analysed therapeutic dialogue as a discursive genre through Dialogical Discourse Analysis. While these contributions have advanced understanding of collaboration at a declarative or structural level, less attention has been paid to how power is interactionally and narratively produced in the situated practices of recovery‐oriented relationships, and specifically to the gap between how collaboration is described by participants and how it is actually enacted in therapeutic dialogue. In therapeutic relationships, power shapes what can be said, heard and transformed. This fluid, multidirectional understanding of power challenges simplistic notions of ‘empowerment’ that assume power can simply be transferred from professionals to service users, and calls instead for attention to how power circulates within therapeutic relationships and the broader institutional and social contexts in which they are embedded.

## 2 Research Questions and Study Significance

The study was guided by three research questions:

1. How are power dynamics enacted within recovery‐oriented therapeutic relationships identified by service users as significant in their recovery trajectories?
2. How do participants’ accounts of collaboration, reciprocity and parity correspond to, or diverge from, the interactional patterns observed in dyadic interviews?
3. How do different facilitator roles – including peer support workers, mental health professionals and family members – relate to the distribution of narrative authority, reciprocity and control within the dyads?

Alongside these empirical questions, the study also pursued a methodological aim: to examine the potential of dyadic interviews and narrative‐interactional analysis for making visible the situated ways in which power is negotiated, shared or asymmetrically distributed within therapeutic relationships.

Central to our conceptual framework is the conceptualisation of power as a continuum rather than a binary.

The theoretical framework integrates Gubrium and Holstein's [14] narrative analysis, Gergen's [12, 13] concept of generative dialogue and Plummer's [15, 16] concept of power flow, converging in an understanding of power as a fluid, negotiated process operationalised through a collaboration‐control continuum. Drawing on these complementary perspectives, we examine power dynamics as ranging along a spectrum from collaboration to control. The collaborative dimension investigates whether interlocutors negotiate, complete, correct or confirm each other's contributions, revealing the degree of narrative equity. Conversely, the control dimension identifies who holds the authority to define, direct or limit the narrative by examining patterns of interference, denial and initiative‐taking. These analytical indices enable researchers to uncover implicit power structures and determine which stories are emphasised or silenced within conversational exchanges.

## 3 Method

This study employed a qualitative interview design to investigate power dynamics within recovery‐oriented therapeutic relationships. It was conducted as part of the project “Exit Madness: Examining Interpersonal Testimonies of Recovery from Severe Mental Disease”, which aimed to explore the relational ingredients of helping relationships considered most important and effective during favourable recovery pathways following severe mental distress.

### 3.1 Participants

This study included 15 dyads, each consisting of a former service user and a facilitator identified by that user as significant to their recovery. Users met the following criteria: (1) past mental disorder diagnosis; (2) no relapse episodes in the preceding 5 years; and (3) subjective perception of change and improvement. These criteria ensured that participants had achieved stable recovery and could reflect on facilitating relationships. Facilitators were user‐identified rather than researcher‐selected, ensuring that the relationships studied were those considered significant by users themselves.

The 15 service users (aged 26–74, mean age = 49) had diagnoses including bipolar disorder (*n* = 6), depression and/or anxiety (*n* = 2), psychosis (*n* = 2), substance use disorder (*n* = 1), and unspecified diagnoses associated with a range of symptoms (n = 4). Facilitators, whose professional roles included peer support workers (PSWs), educators and psychiatrists, were aged between 41 and 63 years (mean age = 51).

All participants (Table 1) were recruited through the collaboration of a recovery‐oriented mental health service in Northern Italy, which acted as a gatekeeper and facilitated initial contact with potentially eligible service users. The service is explicitly oriented towards the principles of recovery, collaborative practice and the recognition of experiential knowledge. A purposive sampling strategy was adopted, with participants selected on the basis of predefined inclusion criteria aligned with the aims of the study.

**Table 1:** Participant characteristics.

Dyad^a^	User diagnosis/experience^b^	Facilitator role
Paolo‐Eugenio	Bipolar disorder	PSW
Benedetta‐Romina	Psychosis, bipolar disorder	PSW
Geremia‐Marco	Bipolar disorder	Brother
Carlo‐Ilaria	Bipolar disorder	Mental health support worker
Brian‐Micaela	Loss of self‐esteem, motivation	Educator
Sandra‐Rita	Emotional instability, isolation	Psychologist
Osvaldo‐Manuela	Depression, bipolar disorder	Mental health support worker
Gianluca‐Sara	Bipolar disorder	Sister
Igor‐Ornella	Emotional distress	Mental health support worker
Piero‐Gennaro	Bipolar disorder	Psychiatrist
Martina‐Filippo	Hallucinations, persecution	Psychologist
Federico‐Giuseppe	Substance‐use disorder	Psychologist
Veronica‐Raffaella	Depression, anxiety	Mental health support worker
Armando‐Monica	Depression, sleep medication dependency	Mental health support worker
Barbara‐Carlo	Hallucinations, psychosis, paranoia	Service coordinator

^a^ All names are pseudonyms.

^b^ Diagnoses and experiences are reported as described by participants, reflecting both clinical and experiential accounts.

As part of the recruitment procedure, service users were asked to identify one person – either within or outside the mental health service – whom they perceived, in retrospect, as having been most instrumental in their recovery process. The 15 individuals identified represented a heterogeneous range of professional and non‐professional roles: peer support workers (*n* = 2), family members (*n* = 2), psychologists (*n* = 3), mental health workers (*n* = 5), a psychiatrist (*n* = 1), an educator (*n* = 1), and a service coordinator (*n* = 1). Importantly, all nominated recovery facilitators agreed to participate in the research through both individual interviews and joint interviews conducted as dyads with the service users who had selected them.

This sampling strategy was adopted because the study aimed to analyse relationships considered significant by service users themselves, rather than relationships selected by professionals or researchers. However, this choice introduces a potential selection bias: the sample likely includes relationships remembered as helpful, positive, or transformative, while less successful, conflictual, or harmful relationships remain outside the analytical field. The findings should therefore be interpreted as about power dynamics within positively identified recovery relationships, and not as representative of therapeutic relationships more broadly.

### 3.2 Data Collection Procedures

Data were collected in two phases using semi‐structured interviews.

#### 3.2.1 Phase One: Individual Interviews

In the first phase, service users and facilitators were interviewed separately to create an independent space for personal expression. This approach allowed each participant to speak freely without the presence of the other, thereby potentially eliciting perspectives or experiences they might not have shared in a joint interview. Table 2 summarise the aims and the questions that make up the individual interviews.

**Table 2:** Aims and questions that make up the individual interviews.

**Service user interview**: The interview explored participants’ recovery journeys and the role of significant relationships in this process. Key questions included: Can you tell me about your journey, from the time of greatest difficulty up to today? What meaning do you give today to what you went through? Would you describe it as illness, suffering, crisis, life experience, or in another way? What do you feel has changed in your life, in your relationship with yourself, with others, and with the future? Which people, relationships, professional figures or services played a significant role in making this change possible? How did they support you? Through which signs, episodes or concrete transformations did you — and those close to you — recognise that something was changing or that you were moving in the right direction?
**Facilitator interview**: The interview explored facilitators’ perspectives on the relationship and their approach to supporting the service user. Key questions included: Which aspects of your relationship do you consider most distinctive in making this therapeutic relationship useful for the recovery journey? If the other person were to tell this story, which elements do you think they would place at the centre? What did you have to take into account in your professional role in order to support this relationship, both in terms of its possibilities and its potential risks or limits?

#### 3.2.2 Phase Two: Dyadic Interviews

In the second phase, service users and facilitators were interviewed together. The dyadic interview is a relatively recent approach in qualitative research, allowing exploration of a ‘third reality’ emerging from the interaction between two participants [20]. Unlike individual interviews, it promotes the co‐construction of meanings and enables the observation of deeper relational processes [21, 22]. As noted by Bjørnholt and Farstad [23] and Szulc and King [24], it facilitates the collection of richer data and the direct observation of interactions while reducing social desirability bias. This dyadic interview format was central to the study's methodology, as it allowed researchers to observe directly how the dyad interacted in constructing a shared narrative about the relationship and the recovery process. All interviews were audio‐recorded and transcribed verbatim. Interviews lasted between 60 and 90 min. Researchers took field notes during and after interviews, documenting non‐verbal communication, emotional tone and interactional dynamics that might not be fully captured in transcripts. Table 3 summarise the aims and the questions that make up the dyadic interview.

**Table 3:** The dyadic interview.

**Questions were designed in three categories:**
Cross‐questions involved role reversal, asking each participant to speak about the other's experience, for example: ‘What did the other person say about the journey at its beginning?’ and ‘How do you think the other person experienced this relationship?’
Individual questions explored each participant's personal experience within the relationship, addressing the same theme from opposite perspectives, for example: ‘What did you have to take into account in your role within the relationship?’ and ‘What challenges did you face in this relationship?’
Dyadic questions addressed the dyad as a single unit, for example: How would you describe the construction of your shared recovery journey, from the initial moment to the identification of a common direction? Which aspects of your relationship made change possible, sustained it, or protected it over time? Through which signs, episodes or concrete transformations did you recognise that change was taking place and that the journey was moving in the right direction? This structure was designed to elicit different types of narrative interaction. Cross‐questions encouraged participants to express empathy and engage with the other's perspective. Individual questions allowed potentially different experiences to be articulated while maintaining focus on the same relational themes. Dyadic questions explicitly positioned the dyad as a unit and encouraged collaborative narrative construction.

#### 3.2.3 Research Positionality and Reflexivity

Researchers introduced themselves to participants as psychologists and psychotherapists, as well as qualitative researchers with doctoral‐level training in the social sciences. Although no formal interviewer training protocol was administered before data collection, the three researchers brought extensive relevant expertise to the interviews: their clinical background as couple and family therapists provided substantial experience in facilitating dyadic conversations and managing interactional dynamics within two‐person exchanges, while their academic training included advanced qualitative methodology and specialised training in narrative and autobiographical analysis.

The study is situated within a social constructionist epistemology, in which the interview is conceived as an interactive space where meanings are co‐produced through the relationship between participants and researchers (Riessman, 2008). Within this framework, reflexivity occupies a central role, requiring explicit attention to how the researchers’ presence and positioning contributed to the production of the narratives collected. This orientation privileged attention to how meanings of suffering and recovery are constructed and negotiated within interaction, rather than foregrounding diagnostic or symptom‐focused readings. None of the researchers had personal experience as a service user within mental health services, nor did any of them hold an institutional affiliation with the service involved in the study. This positioning ‐ external to the service yet clinically and academically informed ‐ is acknowledged as a relevant aspect of the researchers’ reflexive stance, shaping both the conduct of the interviews and the interpretive process.

In the dyadic interviews specifically, the interviewer's role progressively shifted towards that of a moderator of the exchange between the two participants. The dyadic setting frequently acquired a specific significance for participants, functioning as an occasion for shared re‐elaboration of the relational journey and, in some cases, as a moment of symbolic recognition of the therapeutic relationship and its value in the recovery process. As the interactional dynamic between the two participants became central to the unfolding of the narrative, the interviewer gradually assumed a more peripheral position ‐ present in the background of the interactional scene and intervening primarily to support the continuity and balance of the dialogue rather than to direct its content.

### 3.3 Analytical Framework

#### 3.3.1 Gubrium and Holstein's Approach to Narrative Interaction

The analytical approach adopted in this study was based on Gubrium and Holstein's [14] framework for analysing narrative interaction. Unlike traditional conversation analysis, which focuses on the microstructures of interaction, Gubrium and Holstein's approach broadens the analytical lens to understand narratives as situated accomplishments, negotiated and rooted in broader sociocultural contexts.

Their approach involves analysing:

1. How narratives are activated, composed, performed and negotiated in the research context, including who initiates narratives, how they are structured and presented, and how they are responded to within the dyad.
2. The role of the interviewer and research context in co‐constructing narratives. Rather than treating the interviewer as a neutral collector of pre‐existing narratives, this approach recognises that narratives are co‐constructed in the interview interaction itself.
3. Connections between narratives and the broader social and cultural context, including how narratives produced in interviews relate to broader discourses about mental illness, recovery, professional roles and therapeutic relationships.
4. Dynamics of power, agency and collaboration in narrative production, including who has the authority to define, direct or limit the narrative, and how this authority is exercised or resisted.

This approach provides an analytical tool for exploring connections between narratives produced in interviews and the lived realities outside the research context, including the broader social, cultural and power contexts in which they are rooted.

#### 3.3.2 Analytical Codebook: Structure, Codes, and Application Examples

The analysis adopts a hybrid coding approach (Fereday & Muir‐Cochrane, 2006), combining deductive codes drawn from Gubrium and Holstein's [14] six key constructs ‐ Activation, Linkages, Composition, Performance, Collaboration and Control ‐ with inductively generated codes emerging from the data and grounded in Gergen [13]. This dual‐source development yielded a final set of 25 codes, systematised in a codebook to ensure definitional consistency and replicability across analysts, and subsequently applied through ATLAS. ti.

Code development followed an iterative process: an initial deductive phase identified codes derived from the theoretical framework; a subsequent inductive phase integrated new codes emerging from observed patterns not captured by pre‐existing categories. Codes were progressively refined ‐ added, modified or removed ‐ until analytical saturation was reached. Table 4 summarise the elements that make up the analytical codebook.

**Table 4:** Analytical codebook: the constructs definition, the codes and some application examples.

Code group	Construct	Definition	Codes	Applied example
1	Activation: who introduces a narrative topic and why	Captures the mechanisms through which narrative themes are triggered, elicited or self‐generated within the interactional context.	activation‐interviewer; activation‐facilitator; activation‐user; self‐activation‐facilitator; self‐activation‐user.	Paolo (the user) spontaneously introduces ‘recovery as giving help rather than seeking it’ without any external prompt; this was coded as self‐activation‐user and interpreted as an indicator of narrative agency.
2	Linkages: connections between narratives	Identifies expansions of meaning whereby a previously activated theme is enriched through new information or reframing by either participant.	link‐facilitator; link‐user.	Eugenio (the facilitator) connects the dyad's shared similarity to their near‐simultaneous entry into the mental health centre, offering a new interpretive layer; this was coded as link‐facilitator.
3	Composition: how the narrative is structured	Examines the organisational strategies used to construct a coherent narrative, including patterns, metaphors, examples and sequencing.	composition, with a descriptive annotation specifying the observed strategy.	Paolo frames his answer with a contextual introduction before responding directly; this was coded as composition: preliminary framing strategy.
4	Performance: how the narrative is enacted	Captures the situated and embodied dimensions of storytelling: tone, rhythm, pauses, humour and formality.	performance, with a descriptive annotation specifying the observed feature and cross‐referencing with audio recordings.	Eugenio closes a passage with a humorous remark; this was coded as performance: humour, signalling a relaxed atmosphere and relational complicity within the dyadic setting.
5	Collaboration: co‐construction of narrative	Maps the cooperative dynamics through which meaning is jointly built; linked to the Flow of Power continuum toward parity.	collaboration; negotiation; correction‐facilitator/user; completion‐facilitator/user; confirmation‐facilitator/user.	Paolo completes Eugenio's unfinished sentence; this was coded as completion‐user. Eugenio confirms and expands Paolo's account; this was coded as confirmation‐facilitator and completion‐facilitator. High collaboration indices position the dyad toward parity.
6	Control: narrative power and scripting authority	Identifies asymmetric dynamics in which one participant directs, interrupts or restricts the narrative; linked to the Flow of Power continuum toward dominance.	control‐facilitator/user; interference‐facilitator/user; denial‐facilitator/user; initiative‐facilitator/user.	Eugenio responds first to a question addressed to both participants without negotiation; this was coded as initiative‐facilitator and considered a control indicator only if recurrent across the interview.

#### 3.3.3 Analytical Procedure, Triangulation and Reliability

To ensure methodological rigour and inter‐rater reliability, the analytical procedure was structured in several steps and incorporated a two‐level triangulation process (see Table 5).

**Table 5:** Analytical procedure.

Step	Procedure and actions
1	Code development and coding procedure. Codes were developed through the hybrid process described above and documented in the codebook prior to systematic application.
2	For each interview, the same analytical procedure was followed: an initial reading of transcripts and listening to recordings; a content‐focused second reading; a third reading applying the 25 codes from the codebook; identification of macro‐themes via Activation and Linkages codes; and organisation of findings into a five‐quadrant analytical table capturing: (i) dyad description, (ii) dyad in action, (iii) description‐action alignment, (iv) analyst observations, and (v) narrative‐relational dynamic.
3	Inter‐rater reliability. At the coding level, coded transcripts were independently reviewed by secondary analysts not directly involved in the primary coding process. Discrepancies were discussed and resolved through collective revision, establishing inter‐rater agreement across code applications and ensuring coding reliability across the dataset.
4	Derivation of categories. The categories of Parity and Dominance were not imposed a priori but derived through a systematic description‐action alignment procedure: for each dyad, the self‐reported relational description provided by participants was compared against the interactional dynamics observed during the co‐construction of the shared narrative. The balance between Collaboration and Control codes — mapped onto a dominance–parity continuum — informed the final assessment of each dyad's narrative‐relational dynamic. Convergence or divergence between self‐description and turn‐taking interaction provided an additional interpretive layer, strengthening the validity of the categorical assignment.
5	Analytic triangulation. This dual triangulation — across raters and across analytical levels — reduces the risk of single‐analyst interpretive bias and strengthens the overall validity of the qualitative findings.

### 3.4 Cross‐Case Analysis

After completing individual dyad analyses, researchers conducted a cross‐case analysis to identify patterns across dyads. This involved:

1. Categorisation of dyadic dynamics: based on the alignment between described and observed dynamics, dyads were categorised into distinct patterns of narrative‐relational dynamics. These categories emerged from the data rather than being predetermined, although they were informed by the theoretical framework of the collaboration‐control continuum.
2. Role analysis: the study examined whether particular professional roles (peer support worker, psychologist, psychiatrist, family member, etc.) were associated with particular power dynamics. This involved comparing interaction patterns across dyads grouped by facilitator role.
3. Thematic analysis: researchers identified common themes across dyads regarding factors that participants associated with collaborative relationships, challenges to maintaining collaborative dynamics, how power was negotiated around particular topics or decisions, and the relationship between professional role expectations and actual interaction patterns.
4. Pattern mapping: researchers created visual representations of how dyads were distributed across the collaboration‐control continuum and examined what factors distinguished dyads at different points on the continuum.

### 3.5 Ethical Procedure and Interpretive Validation

Ethical approval was granted by the Ethics Committee of the School of Psychology of the University of Padua (Protocol No. 4862, 30/06/2022). Informed consent was obtained from all participants prior to data collection. Participants were offered the opportunity to receive feedback about the study's findings. This was considered both an ethical obligation and an opportunity to validate interpretations with those who had direct experience of the relationships being studied.

## 4 Results

Category assignment was based on the systematic Description‐Action alignment procedure (see Tables 6, 7, 8, 9) and cross‐validated through inter‐rater discussion. Percentages reflect the distribution across the 15 dyads.

**Table 6:** An example of a parity dyad.

Description of the dyad	Dyad in action	Description‐Action alignment	Narrative‐relational dynamic
Armando and Monica describe their relationship as “strongly reciprocal and human”. […] Armando recognises Monica as a key figure in his process of change, while Monica emphasises that Armando also had an impact on her: “it was an encounter that made us both better”. […] According to Armando, from the beginning Monica was able not to “force” her interventions or impose herself, but rather to stand alongside him, always offering appropriate feedback.	The interviewer's questions often develop into genuine conversations in which the two participants address each other directly and respond through a series of confirmations of the other's words and completions of what the other has introduced, indicating a strong collaborative dynamic. […] Especially toward the end of the interview, it is possible to observe a friendly and playful atmosphere between them, with both participants exchanging jokes and sarcastic comments.	The observation of the interactive dynamics reveals the mutual esteem and reciprocity that both participants identify as characterising their relationship. In many responses, they offer each other compliments and words of affirmation. Reciprocity is also clearly visible […]. The strong affection that connects them is also observable, both in the friendly atmosphere and in specific gestures: for example, toward the end of the interview, Armando gives Monica a handmade gift, using the occasion to thank her and express his gratitude.	Both the narrative and the relationship appear to be egalitarian and collaborative.

**Table 7:** An example of declared parity with observed dominance.

Description of the dyad	Dyad in action	Description–action alignment	Narrative‐relational dynamic
Brian and Micaela describe their relationship as a “good relationship”, characterised by greater intimacy than other therapeutic relationships. […] The relationship predates Micaela's professional role and is grounded in their shared passion for practical activities, mutual trust, and the negotiation and co‐construction of the goals of the pathway.	The narrative is mainly guided by the interviewer's questions. Brian always responds first, but with brief and relatively undeveloped contributions; Micaela systematically intervenes to expand on what he has introduced, taking the lead in the discourse. […]	The narrative co‐construction described by the dyad does not fully correspond to the observed practice. The discourse is dominated by Micaela, although it develops from the cues introduced by Brian. […]	Micaela dominates the narrative while remaining aligned with the themes introduced by Brian. However, the egalitarian relationship described by the dyad is not reflected in the observed narrative dynamic. Despite both participants’ collaborative descriptions, the observed asymmetry reveals a gap between declared parity and interactional practice.

**Table 8:** An example of non‐categorizable dynamics.

Description of the dyad	Dyad in action	Description–action alignment	Narrative‐relational dynamic
The relationship between Carlo and Ilaria developed over time, evolving from an initial service user–mental health practitioner relationship into a more equal and intimate connection once Carlo took on the role of PSW. […]. Carlo, in fact, recounts that in his relationship with Ilaria he felt “not just a service user”, but rather experienced himself as a person with his own identity, sharing with her not only his lived experience, but also his thoughts and desires. Ilaria welcomed these forms of sharing with openness and availability, while always respecting the boundaries imposed by their professional roles.	Both collaborative and controlling aspects can be identified in the narrative. At the compositional level, there is an initially more individualistic narrative tendency in the discussion of the first themes, which gradually becomes more shared when addressing the final topics. […]. The narrative is initially dominated by Carlo, and later by Ilaria. […]. Toward the end of the interview, Carlo and Ilaria begin to alternate more frequently in narrating the same story, thereby co‐constructing it.	In parallel with the narrative modalities, the content of their account also reveals moments in which their relationship was initially dominated by one member and later became more egalitarian, in relation to the role Carlo occupied across the different phases of the pathway.	At the time of the interview, the relationship can be described as egalitarian, whereas the narrative shows levels of collaboration that are not sufficiently high to speak of a predominantly co‐constructed account or of a sufficiently developed generative dialogue. […]. Control elements are predominant, although distributed between both participants.

**Table 9:** An example of a dominance dyad.

Description of the dyad	Dyad in action	Description–action alignment	Narrative‐relational dynamic
Igor and Ornella describe their relationship as characterised by trust, mutual esteem, and shared growth. […] Ornella defines herself as “directive”, determined and firm, while seeking to balance rigour and support. Igor recognises her ability to motivate him and encourage him to remain consistent. […] Ornella's capacity to guide Igor without imposing on him is acknowledged.	The narrative is predominantly controlled by Ornella, who takes the floor more often than Igor and directs his discourse through invitations to narrate specific episodes. […]. Igor's completion interventions often consist of repeating what Ornella has already said, using different words and adding only minor details; as a result, they function more as confirmations than as genuine completions. […] The two interact throughout the interview, but their dialogue cannot be described as particularly generative.	Ornella's described “directiveness” can be observed in the way she positions herself during the narrative with Igor. She tends to maintain control of the conversation and to ask for his opinion on what has been said, thereby limiting him to a confirmatory role, or guiding him toward a story that she wants him to address. […]	The narrative can overall be understood as dominated by Ornella. The relationship is described as predominantly directed by Ornella, despite Igor's recent acquisition of autonomy. […] Both the relationship and the narrative appear to be dominated by Ornella.

### 4.1 Overview of Findings

Parity (9 dyads, 60%): relationships characterised by collaborative narrative construction, relatively balanced power distribution and strong alignment between described and observed dynamics.

Declared parity with observed dominance (3 dyads, 20%): relationships described by participants as collaborative and egalitarian, but in which interactional patterns suggested more asymmetrical or controlling dynamics.

Non‐categorizable dynamics (2 dyads, 13%): relationships that could not be meaningfully categorised according to the criteria used, often due to unique or complex patterns that did not fit the established categories.

Dominance dynamics (1 dyad, 7%): a relationship characterised by more controlling dynamics that were acknowledged by both participants.

These categories should not be understood as rigid classifications, but rather as indicative of the principal characteristics of power flow within each dyad. Their boundaries are permeable, and individual dyads may display features of multiple categories at different moments or around different topics.

### 4.2 Detailed Analysis of Narrative‐Relational Dynamics

#### 4.2.1 Parity Dynamics (9 Dyads, 60%)

These relationships are characterised by collaborative narrative construction, relatively balanced power distribution and strong alignment between described and observed dynamics. These dyads engaged in what Gergen [12, 13] terms ‘generative dialogue’: communicative interaction in which participants collaboratively co‐created new possibilities of meaning. This was evident through:

Frequent mutual recognition and affirmation: participants regularly expressed appreciation for each other's contributions, both to the relationship and to the narrative being constructed in the interview. This was evident through statements of appreciation and gratitude, confirmation and validation of the other's perspective, building upon rather than contradicting the other's contributions, and non‐verbal indicators of engagement and affirmation.

Narrative completion and enrichment: rather than one participant dominating the narrative while the other simply confirmed, parity dyads engaged in reciprocal completion and enrichment of narratives. Both participants actively contributed to developing stories, adding details, perspectives and interpretations that created a richer, more multifaceted account than either could have provided alone.

Fluid power flow: in parity dyads, the ‘flow of power’ [15, 16] was notably fluid and flexible. While one participant might take the lead in narrating a particular episode or theme, this leadership was temporary and frequently shifted. Neither participant consistently controlled the narrative direction or constrained the other's contributions.

Humour and ease: many parity dyads cultivated a climate of ease and humour, with participants exchanging jokes and playful comments. This levity appeared both to reflect and to reinforce the collaborative nature of the relationship, creating a space in which both participants felt comfortable expressing themselves freely.

Reciprocity in description and action: a defining characteristic of parity dyads was the strong alignment between how participants described their relationship and how they actually interacted. When participants described their relationship as collaborative, reciprocal or egalitarian, these characteristics were clearly observable in their interaction patterns.

Other factors associated with parity dynamics:

Explicit commitment to collaboration: facilitators in parity dyads often articulated an explicit commitment to collaborative approaches, describing their role as supporting rather than directing, accompanying rather than leading.

Recognition of multiple forms of expertise: parity dyads were characterised by mutual recognition that both participants brought valuable knowledge and expertise: professionals bringing training and clinical experience, and users bringing lived experience and intimate self‐knowledge.

Time and relationship development: many dyads described how their relationship had evolved over time, often beginning with more traditional professional‐patient dynamics and gradually developing greater equality as trust developed and the user's recovery progressed.

Shared values and goals: parity dyads frequently described having developed a shared understanding of recovery goals and values, with both participants working toward mutually agreed objectives rather than the professional imposing goals on the user.

Reflexivity about power: facilitators in parity dyads often took a reflexive stance on power dynamics, describing conscious efforts to minimise professional authority and create space for the user's voice.

#### 4.2.2 Declared Parity with Observed Dominance (3 Dyads, 20%)

Three dyads were interpreted as reflecting a pattern in which participants described their relationship as collaborative and egalitarian, but observed interaction revealed more asymmetrical power dynamics. This discrepancy between description and action represents a particularly important finding, highlighting potential gaps between rhetoric and everyday practice.

Characteristics of declared parity with observed dominance dyads:

Participants described relationships characterised by collaboration, partnership and shared decision‐making, yet interactional patterns often leaned toward more directive or controlling approaches.

Less fluid power flow: unlike parity dyads, where power flowed fluidly between participants, these dyads showed a more uneven distribution of influence, with one participant – sometimes the facilitator, sometimes the user – more consistently controlling narrative direction and content.

Disagreement about reciprocity: in some cases, participants showed subtle disagreement about the degree of reciprocity in their relationship. While both might describe the relationship as collaborative, closer examination revealed different understandings of what this meant, with one participant perceiving more equality than the other experienced.

Greater balance between control and collaboration codes: while parity dyads showed a predominance of collaboration codes over control codes, these dyads showed a more balanced distribution, with substantial instances of both collaborative and controlling practices.

Presence of conflictual elements: some dyads in this category included subtle conflictual elements ‐ moments of disagreement, correction or tension that were less evident in parity dyads. These conflicts were typically mild and quickly resolved, but their presence suggested underlying power dynamics that complicated any simple characterisation as collaborative.

Factors associated with declared parity with observed dominance:

Professional role expectations: in some cases, traditional professional role expectations appeared to structure interaction despite conscious commitment to collaborative approaches. Professionals trained in directive therapeutic models might unconsciously enact these patterns even when explicitly endorsing collaborative principles.

Stage of recovery: dyads in this category were characterised by users being at earlier stages of recovery, potentially requiring more directive professional involvement. However, this raises important questions about whether such directiveness is genuinely necessary or reflects professional assumptions about user capacity.

Lack of awareness of power dynamics: in some cases, participants appeared genuinely unaware of power asymmetries in their interaction, suggesting that awareness of power dynamics cannot be assumed even in recovery‐oriented contexts.

Institutional constraints: some facilitators described institutional or organisational constraints that limited their ability to practise as collaboratively as they wished, suggesting that individual commitment to collaboration may be insufficient without supportive organisational structures.

#### 4.2.3 Non‐Categorizable Dynamics (2 Dyads, 13%)

Two dyads could not be meaningfully categorised according to the criteria used, owing to unique or complex patterns that did not fit the established categories. Rather than forcing these dyads into inappropriate categories, they were identified as non‐categorisable, highlighting the limitations of any categorical system for capturing the full complexity of power dynamics (see Table 8). The analysis of these two dyads illustrates how power dynamics evolve over time and how past configurations continue to influence present interactions. Power dynamics may vary substantially depending on what is being discussed, suggesting that power is not a global characteristic of relationships but may be topic‐ or context‐specific. Specifically, the non‐categorizable dyads highlight the temporal and biographical evolution of power. For instance, the transition from service user to ‘Expert by Experience’ (peer worker) creates a hybrid relational space where past hierarchies and present collegiality coexist. These cases illustrate that power flow is not only situational but also historical; the ‘ghosts’ of previous clinical roles may continue to influence narrative agency even after a formal shift in status.

#### 4.2.4 Dominance Dynamics (1 Dyad, 7%)

One dyad was interpreted as reflecting what we categorised as ‘dominance’ dynamics, characterised by more controlling patterns that were acknowledged by both participants. The dominance observed in the Igor‐Ornella dyad challenges a binary view of power as inherently repressive. When directiveness is transparently acknowledged and aligned with the user's current recovery stage, it functions as scaffolding rather than as imposition. This shifts the clinical focus from the achievement of total parity to the ensuring of power transparency.

Characteristics of dominance dyads:

Acknowledged asymmetry: unlike declared parity with observed dominance dyads, both participants explicitly acknowledged power asymmetry. Ornella described herself as directive, and Igor confirmed this characterisation. This transparency distinguishes dominance from unrecognised control.

Consistent control pattern: the facilitator consistently controlled narrative direction, initiated topics and constrained user contributions. This pattern was stable throughout the interview rather than shifting between participants.

Confirmatory rather than co‐constructive user role: the user's contributions primarily confirmed or minimally elaborated the facilitator's narratives, rather than introducing new perspectives or directions. Igor's completions were essentially repetitions with slight variations rather than genuine additions.

Limited generative dialogue: although interaction occurred throughout, it lacked the generative quality of collaborative dyads. New meanings, understandings or perspectives did not emerge from the dialogue itself.

Directive invitation to narrative: the facilitator directed which stories should be told and when, using questions and invitations that constrained rather than opened narrative possibilities.

Experience as helpful despite control: critically, Igor experienced Ornella's directiveness as motivating and helpful for his recovery. He valued her capacity to push him toward consistency and goal achievement, suggesting that acknowledged directiveness serving user‐defined goals may be experienced positively.

## 5 Power Dynamics and Professional Roles

Power dynamics did not correlate in a predictable manner with professional roles. Analysis across facilitator roles revealed:

Some professional roles (e.g., psychologist, Mental Health Support Worker) were associated with different power dynamics across dyads. No professional role consistently produced either collaborative or controlling dynamics. This challenges assumptions that particular roles inherently produce particular power configurations, suggesting that dynamics are shaped by specific relational and interactional practices rather than role expectations alone.

## 6 Discussion

This study explored how power dynamics are interactionally enacted within recovery‐oriented therapeutic relationships described by service users as meaningful. The findings suggest that collaboration should not be understood as a stable relational property, nor as a direct consequence of the recovery‐oriented values formally espoused by services. Rather, collaboration emerges as an interactional achievement: something that is produced, sustained, or constrained through situated practices of turn‐taking, narrative completion, confirmation, redirection, and mutual recognition.

This interpretation is consistent with narrative and social constructionist perspectives, which understand meaning and relational positioning as co‐produced in interaction. Nevertheless, the findings should be read as an interpretive account of a small qualitative sample, oriented toward in‐depth understanding of the dyads examined rather than statistical generalisation. The configurations observed are not intended as evidence of the prevalence of specific power arrangements in recovery‐oriented services, but as conceptual tools for making visible interactional dynamics that are otherwise difficult to capture through conventional outcome‐based assessments.

No dyad could be understood as exclusively collaborative or controlling; instead, each reflected shifting configurations in which both elements were present in different ways. This reading resonates with Plummer's [15, 16] concept of ‘power flow’ and challenges binary categorisations of relationships as either collaborative or paternalistic. This fluidity has important implications. Theoretically, it aligns with social constructionist perspectives that emphasise power as continuously constructed through interaction [12, 13, 14] and with Foucault's [25] conceptualisation of power as circulating through relationships, both constraining and enabling rather than purely repressive. Practically, it suggests that evaluating collaboration requires attention to patterns over time rather than to single moments, and invites recognition that certain forms of directiveness may coexist with genuine partnership.

The 60% of dyads interpreted as reflecting parity‐oriented dynamics may be read as encouraging, suggesting that recovery‐oriented approaches can facilitate genuinely egalitarian relationships, supporting Recovery Movement aspirations [26, 27] and the WHO's emphasis on user empowerment. The analysis revealed specific observable practices characterising collaboration: generative dialogue creating new meanings [12, 13], mutual recognition and affirmation [28], reciprocal narrative completion [14], fluid power flow, and relational ease with humour [29]. These concrete indicators move beyond abstract principles to teachable patterns. Facilitating factors included explicit commitment to collaboration, recognition of multiple forms of expertise, shared lived experience in peer relationships, relationship development over time, organisational support, and reflexivity about power. However, explicit commitment alone proved insufficient: some facilitators in declared parity with observed dominance dyads articulated collaborative principles without enacting them. This suggests that awareness and intention must be accompanied by specific interactional skills and ongoing self‐examination, aligning with critical perspectives on practitioner reflexivity [30, 31]. Both peer support dyads revealed parity, suggesting that shared lived experience may facilitate egalitarian dynamics through reduced status differentials and shared understanding. However, some professional relationships also highlighted strong collaboration, indicating that shared experience facilitates, but does not guarantee, collaboration. This supports growing evidence on peer support effectiveness [32, 33], while recognising that both professional training and lived experience can contribute to collaborative practice.

The 33% of dyads showing declared parity with observed dominance represents a significant concern, highlighting gaps between recovery rhetoric and practice. This aligns with research documenting tensions between recovery principles and implementation [5, 6] and suggests a rhetorical trap in which the language of recovery is adopted as professional nomenclature without a corresponding shift in interactional habits. Traditional clinical power structures may therefore remain deeply entrenched and operate as an invisible default, even when practitioners consciously commit to egalitarian principles. Several factors contributed to this gap. Professional role expectations may structure interaction despite conscious collaborative commitment, with practitioners trained in directive models unconsciously enacting these patterns [34]. Institutional constraints ‐ time pressures, documentation requirements, risk protocols and hierarchical cultures ‐ limited collaborative practice despite individual commitment, indicating that transformation requires systemic change rather than individual training alone [6, 35]. Furthermore, some participants appeared genuinely unaware of controlling patterns evident to researchers, underscoring the importance of feedback mechanisms that allow practitioners to examine actual interactional practices rather than relying only on self‐perception. Interestingly, one dyad (Osvaldo‐Manuela) showed user rather than facilitator dominance, challenging simplistic assumptions that empowering users automatically produces collaboration and confirming that genuine collaboration requires balanced participation from both parties.

Power dynamics did not follow predictable patterns linked to professional roles (Table 10), inviting reconsideration of assumptions underlying much mental health policy and practice. Traditional approaches often operate on the premise that particular roles inherently produce particular power configurations: psychiatrists as necessarily more authoritarian than psychologists, or peer support workers as necessarily more egalitarian than professional staff. Our findings suggest that these assumptions are overly simplistic. While role expectations and training may influence interaction, they do not determine it. Power dynamics emerge from specific relational and interactional practices, situated within broader institutional and socio‐historical structures, rather than being predetermined by role categories. The lack of a clear link between professional roles and relational patterns challenges the essentialist view that certain disciplines are inherently more authoritarian than others, shifting the analytical focus from who the facilitator is (role‐based power) to how the facilitator acts (interaction‐based power). Collaborative competence thus emerges as a cross‐disciplinary skill that transcends formal training and depends on the specific relational ethos developed within the dyad. This aligns with positioning theory [36] and resonates with therapeutic alliance research emphasising relationship quality over therapist characteristics or theoretical orientation [37].

**Table 10:** Professional roles and power dynamics.

Professional role	Characteristic of the dyad
Peer Support Workers (2)	Both were interpreted as reflecting parity.
Family Members (2)	Both were interpreted as reflecting parity.
Psychologists (3)	Two were interpreted as reflecting parity; one as declared parity with observed dominance.
Mental Health Support Worker (5)	Two were interpreted as reflecting parity; one as declared parity with observed dominance; one as non‐categorizable; and one as dominance.
Psychiatrist (1)	Non‐categorizable.
Educator (1)	Declared parity with observed dominance.
Service coordinator (1)	Parity.

The single dominance dyad (Igor‐Ornella) warrants particular attention. The relationship was experienced as effective despite ‐ or perhaps because of ‐ its directive elements, suggesting that some degree of directiveness may be appropriate in certain recovery contexts, particularly at earlier stages when users may benefit from greater structure. Critically, this directiveness was acknowledged and valued by both participants, and Igor described gaining increasing autonomy over time. This consensual directiveness appears to function as co‐constructed scaffolding for recovery rather than as unilateral imposition: its therapeutic value lies not in control itself, but in the alignment between the facilitator's guidance and the user's self‐defined goals at specific phases of the recovery journey. These findings point to the contextual and relational nature of therapeutic effectiveness. The crucial question is not whether directiveness is present or absent, but whether it is transparent, negotiable, responsive to the user's current recovery needs, open to disagreement without negative consequences, and oriented toward user‐defined rather than institutional goals. They also align with research emphasising responsiveness to individual preferences over rigid adherence to particular therapeutic models [38, 39].

### 6.1 Implications for Practice

Fostering genuinely collaborative therapeutic relationships requires more than endorsing recovery principles: it demands the development of specific interactional skills, including the creation of space for the user's voice, building upon rather than redirecting user contributions, recognising experiential knowledge, and negotiating rather than imposing meanings. Training should include opportunities to observe and practise these skills through feedback on actual interaction patterns. Equally essential is the cultivation of reflexivity about power ‐ the capacity to examine one's own interactional practices and their relational effects ‐ supported by ongoing supervision and peer consultation focused on power dynamics rather than on clinical content alone. To move beyond declared parity, mental health services should consider implementing interactional supervision using audio or video recordings to provide practitioners with objective feedback on narrative control patterns.

Individual commitment, however, is insufficient without supportive organisational structures. Organisations must examine how policies, procedures and cultures either enable or constrain collaborative practice, including time allocation for genuine dialogue, documentation systems, risk management approaches and hierarchical structures that may inadvertently reproduce power asymmetries. Organisational cultures should actively support the relational ease and generative dialogue identified as hallmarks of parity dyads, and create mechanisms for the ongoing examination of power dynamics through user feedback systems and open forums. Peer support workers and users with lived experience should be involved not only in individual care but also in organisational governance and policy development.

Service evaluation should extend beyond outcomes and user satisfaction to include attention to power dynamics in therapeutic relationships, prioritising observational methods that examine actual interactions and Description‐Action alignment rather than self‐report alone. Finally, given that power dynamics evolve over time and that some individuals may benefit from more directive support at earlier recovery stages, services should support flexible, individually responsive approaches and ensure that therapeutic relationships are regularly reviewed to assess whether power distributions remain appropriate to current recovery needs.

## 7 Conclusions

This study demonstrates that power in recovery‐oriented therapeutic relationships operates as a fluid, negotiated process rather than as a fixed attribute of individuals or roles. The 60% of parity‐oriented dyads suggests that recovery‐oriented approaches can facilitate genuinely egalitarian relationships; yet the 33% showing Description‐Action gaps reveals persistent challenges in translating collaborative rhetoric into practice: a professional blind spot in which the lexicon of recovery is adopted without dismantling the interactional habits of the medical model. Power, our findings suggest, is not merely an ideological choice but an embodied conversational routine that requires deliberate training to be recognised and renegotiated. The absence of a predictable link between professional role and power dynamic further implies that collaborative competence is available to any practitioner, regardless of disciplinary background, provided that appropriate training, organisational support and commitment to reflexivity are in place.

As Foucault reminds us: “The problem is not to try to dissolve [power relations] in the utopia of completely transparent communication, but to acquire the rules of law, the management techniques, and also the morality, the ethos, the practice of the self, that will allow us to play these games of power with as little domination as possible” [25]. This study contributes to that project by illuminating how power operates in recovery relationships and by identifying the interactional practices and organisational conditions that can facilitate playing these games of power with minimal domination.

## 8 Limitations

Several limitations warrant consideration. The relatively small sample size (15 dyads) limits generalisability and prevents definitive conclusions about factors associated with different power dynamics. The cross‐sectional design captured relationships at a single time point, potentially missing important temporal dynamics.

The study examined relationships within a single recovery‐oriented service in Italy, limiting generalisability to other contexts. Services with different organisational cultures, resources or levels of recovery‐oriented practice implementation might show different patterns. Cultural factors specific to the Italian context may also influence power dynamics in ways that are not generalisable to other cultural settings.

The dyadic interview format, while providing valuable observational data, is itself an artificial context that may not fully represent how dyads interact in natural therapeutic settings. Participants may have performed their relationships differently in the research context than in everyday practice. Additionally, researcher presence and the interview structure itself inevitably influenced the interactions observed. A further limitation concerns the nomination procedure through which dyads were constituted. By asking service users to identify their most significant recovery relationship, the sample likely over‐represents positive and transformative relationships, while conflictual or discontinued ones remain outside the analytical field. The findings should therefore be interpreted as pertaining specifically to positively identified recovery relationships, and not as representative of the full range of power dynamics in recovery‐oriented services.

The study did not systematically examine potentially important variables, including relationship duration, frequency of contact, specific diagnoses, symptom severity, or demographic factors such as age, gender and socioeconomic status, all of which may influence power dynamics in ways not fully captured by the present analysis. Finally, the study focused on dyadic interactions without examining broader systemic factors ‐ organisational cultures, policies, resource constraints or societal discourses about mental illness and recovery ‐ that shape the possibilities for collaborative practice. While some facilitators mentioned organisational factors, these were not systematically investigated.

Gender configurations within dyads may also have influenced narrative authority and turn‐taking patterns in ways not fully captured by our framework. Future research using larger and more demographically diverse samples would be better positioned to examine how demographic factors intersect with interactional power dynamics in recovery‐oriented relationships.

## Author Contribution

**Elena Faccio:** conceptualization, methodology, funding acquisition, writing – original draft, writing – review and editing, investigation, project administration, supervision. **Michele Rocelli:** investigation, validation, writing – review and editing, data curation, resources. **Lorenzo Binaggia:** methodology, data curation, formal analysis. **Elisa Giolli:** methodology, formal analysis, data curation. **Diego Romaioli:** writing – review and editing, supervision. **Giacomo Chiara:** writing – review and editing, supervision. **Claudio Agostini:** data curation, supervision, project administration. **Roberto Vitelli:** writing – review and editing, validation, supervision. **Ludovica Aquili:** methodology, data curation, resources, formal analysis, writing – review and editing.

## Ethics Statement

Approval for the study was obtained from the Ethical Committee of the School of Padova, at the University of Padova (approval no. 4862 30/06/2022). All patients provided written informed consent prior to enrolment in the study. The participants were informed of their right to withdraw at any time. They signed a written informed consent form regarding their participation in the research and its publication.

## Conflicts of Interest

The authors declare no conflicts of interest.

## Data Availability

Due to the sensitive nature of the data and to protect participant confidentiality, interview transcripts are not publicly available. Anonymized analysis tables and coding summaries may be available from the corresponding author upon reasonable request and with appropriate ethical approval.
